# KPC and NDM-1 Genes in Related *Enterobacteriaceae* Strains and Plasmids from Pakistan and the United States

**DOI:** 10.3201/eid2106.141504

**Published:** 2015-06

**Authors:** Mitchell W. Pesesky, Tahir Hussain, Meghan Wallace, Bin Wang, Saadia Andleeb, Carey-Ann D. Burnham, Gautam Dantas

**Affiliations:** Washington University School of Medicine in St. Louis, St. Louis, Missouri, USA (M.W. Pesesky, T. Hussain. M. Wallace, B. Wang, C.D. Burnham, G. Dantas);; National University of Sciences and Technology, Islamabad, Pakistan (T. Hussain, S. Andleeb)

**Keywords:** Carbapenems, antimicrobial resistance, Enterobacter, Enterobacteriaceae, Escherichia coli, gene transfer, genomics, Klebsiella infections, Klebsiella pneumonia, bacteria, multilocus sequence typing, phylogeny, plasmids, transposases, United States, Pakistan, antibiotic drugs, beta-Lactam resistance, beta-Lactamases, beta-Lactams, next-generation sequencing

## Abstract

To characterize the genomic context of New Delhi metallo-β-lactamase-1 (NDM-1) and *Klebsiella pneumoniae* carbapenemase (KPC), we sequenced 78 *Enterobacteriaceae* isolates from Pakistan and the United States encoding KPC, NDM-1, or no carbapenemase. High similarities of the results indicate rapid spread of carbapenem resistance between strains, including globally disseminated pathogens.

Pathogenic *Enterobacteriaceae*, including *Escherichia coli* and *Klebsiella pneumoniae*, are major causes of multidrug-resistant (MDR) infections in hospitals worldwide. These pathogens have recently been shown to have acquired resistance to carbapenems, and the US Centers for Disease Control and Prevention identified carbapenem-resistant *Enterobacteriaceae* as of the 3 most urgent MDR threats (*1*). Among the *Enterobacteriaceae*, β-lactam resistance, including carbapenem resistance, is primarily caused by enzymatic degradation by β-lactamases. Two carbapenemase subclasses are especially problematic: *Klebsiella pneumoniae* carbapenemase (KPC) and New Delhi metallo-β-lactamase-1 (NDM-1). KPC, identified in 2001 (*2*), has become endemic to several noncontiguous areas of the world, including the United States, Israel, Greece, South America, and China (*3*). NDM-1 was first described in 2008, although retrospective studies identified NDM-1 from 2006 (*4*) and is abundant in New Delhi water samples (*5*). Most patients from whom NDM-1 is isolated have an epidemiologic link to the Indian subcontinent, but NDM-1 has also recently become endemic to the Balkans and Middle East (*6*).

The spread of antibiotic resistance genes such as NDM-1 and KPC is facilitated by horizontal gene transfer (HGT) between bacteria (*7*). Among globally disseminated pathogens, HGT facilitates combination of the most effective antibiotic resistance genes from diverse geographies into multidrug resistance plasmids that spread between strains. Recombination and transposition have created populations of these plasmids that have related architectures but vary in their composition of antibiotic drug resistance cassettes (*8*). This effect has enabled both KPC and NDM-1 to rapidly expand within the *Enterobacteriaceae* and other proteobacterial pathogens, such as *Acinetobacter baumanii* (*9*,*10*). Antibiotic resistance genes can also spread through clonal expansion in successful pathogenic strains, for example, KPC in *K. pneumoniae* sequence type (ST) 258 (*11*), and the extended-spectrum β-lactamase CTX-M-15 in *E. coli* ST131 (*12*). Both HGT and clonal expansion have enabled KPC and NDM-1 to rapidly spread to distant locations after their emergence (*6*,*8*). 

The similarities in the spread and resistance spectra of KPC and NDM-1 (both provide resistance to nearly all β-lactam antimicrobial drugs) leads to the hypothesis that similar mobile elements will make both genes available to similar pathogen populations. We tested this hypothesis by examining clinical *Enterobacteriaceae* isolates from Pakistan and the United States encoding NDM-1, KPC, or no carbapenemase.

## The Study

We collected 450 bacterial isolates (including 195 *Enterobacteriaceae*) in Pakistan during February 2012–March 2013 from Pakistan Railway General Hospital in Rawalpindi and the Pakistan Institute of Medical Sciences in Islamabad. From this collection, we randomly selected 55 *Enterobacteriaceae* isolates for whole-genome sequencing. We then selected 23 isolates from samples collected in the United States during January 2010–June 2013 from patients in Barnes Jewish Hospital in St. Louis, Missouri, that had similar proportions of β-lactam susceptibility and resistance to the isolates collected in Pakistan for sequencing. All isolates were de-identified and retrieved from existing strain banks. The combined set included 33 *E. coli*, 30 *K. pneumoniae*, 9 *Enterobacter cloacae*, and 6 *Enterobacter aerogenes* (Technical Appendix Table 1). We extracted plasmid DNA from 9 isolates encoding NDM-1, 11 isolates encoding KPC, and 3 isolates encoding CTX-M-15 and performed shotgun sequencing on those plasmid preparations. Detailed methods are described in the Technical Appendix.

**Figure 1:**
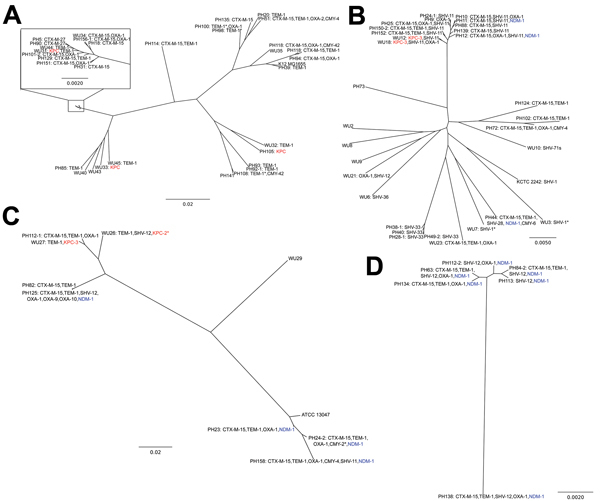
Distribution of antimicrobial drug resistance genotypes of KPC and NDM-1 genes in related *Enterobacteriaceae* strains and plasmids in Pakistan and the United States. Phylogenetic trees have been annotated with the specific β-lactamases encoded by those isolates. *Klebsiella pneumoniae* carbapenemase carriage is indicated by bold text, and New Delhi metallo-β-lactamase-1 carriage is indicated by bold, underlined text. A) *Escherichia coli*; B) *Klebsiella pneumoniae*; C) *Enterobacter cloacae*; D) *Enterobacter aerogenes*. *Denotes an unnamed single nucleotide variant of the named β-lactamase. Scale bars indicate nucleotide substitutions per site.

Using antibiotic resistance gene predictions from the Resfams database (*13*) and core genome alignment, we constructed a phylogenetic tree for each species in our set, overlaid by the β-lactamases encoded by each isolate (Figure 1). Isolates from both locations were found to be members of the same subspecies clades (Technical Appendix Figure 1) and to contain similar repertoires of β-lactamases (Figure 1), indicating that geography is not a discriminating variable for these isolates. Many of these isolates were also MDR: resistance to ciprofloxacin, trimethoprim/sulfamethoxazole, gentamicin, doxycycline, and chloramphenicol occurred in 63%, 65%, 45%, 54%, and 56% of isolates, respectively. As expected from results of previous work (*8*), *E. coli* ST131 isolates had high rates of CTX-M carriage (82%; Figure 1, panel A) and ciprofloxacin resistance (100%).

The variety of strains that we discovered encoding KPC and NDM-1 is consistent with existing evidence that HGT is a major factor in their spread. All KPC genes were proximal to Tn4401 and all NDM-1 genes were carried on ISAba125, mobile elements with which each gene has respectively been previously associated (*14*). We observed multiple examples of NDM-1 within the *K. pneumoniae* ST11 clade (*15*) (Figure 1, panel B; Technical Appendix Figure 1, panel B), a close relative of ST258. This association could be caused by clonal expansion or multiple HGT events and emphasizes that lineages known to encode KPC are now also acquiring NDM-1. We also observed high rates of NDM-1 carriage in *Enterobacter* isolates (Figure 1, panels C and D), which in general showed a high number (maximum 8) and wide variety of β-lactamases. These isolates were also MDR: 57% of the *Enterobacter* isolates were resistant to all or all but 1 of the antimicrobial drugs tested. At best, these *Enterobacter* strains are a reservoir for resistance in Pakistan; at worst, they are the vanguard of an expansion of carbapenem-resistant *Enterobacter* infections.

**Figure 2:**
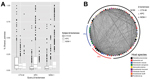
Pairwise BLAST identity (http://blast.ncbi.nlm.nih.gov/Blast.cgi) of all CTX-M genes, *Klebsiella pneumoniae* carbapenemase (KPC), and New Delhi metallo-β-Lacatamase-1 (NDM-1) plasmids from isolates collected in Pakistan and the United States plasmid preparations, and the National Center for Biotechnology Information database complete plasmids. An all-against-all plasmid BLAST was performed and plasmid interactions were defined by the percentage of the query plasmid conserved (at ≥99% identity) in the subject plasmid. A) Plasmid interactions collected based on the defining β-lactamase of their query and subject plasmids. Box and whisker plots represent the range of pairwise sharing values within this population of plasmids. Upper and lower boundaries of the box correspond to the first and thirds quartiles; whiskers (error bars) represent 1.5 times the interquartile range; points beyond the whiskers represent outliers. B) Network map in which nodes represent individual plasmids and lines represent regions shared between plasmids. Line width is proportional to the number of nucleotides contained in fragments >500 bp in length at >99% sequence identity. Genetic elements repeated within the same plasmid DNA are represented by lines that leave and return to the same node. Plasmid sequence origin is indicated in arcs around the network.

Previous observations have predominantly found KPC and NDM-1 to be expressed from plasmids (*6*,*11*). To characterize the sequence similarity of plasmids within the NDM- and KPC-carrying plasmid populations, we purified and sequenced plasmid DNA from 9 isolates encoding NDM-1, 11 encoding KPC, and 3 encoding CTX-M-15. Sequencing showed that these plasmids include representatives from IncHI2, IncY, IncN, IncFIA, IncFIB, IncFIC, and IncI1 incompatibility groups. Using reciprocal BLAST (http://blast.ncbi.nlm.nih.gov/Blast.cgi) alignment between each pair of plasmid preparations, we calculated the percentage of each plasmid shared using a 99% identity threshold. We performed this same analysis for all sequenced plasmids containing NDM-1, KPC, or CTX-M available in the National Center for Biotechnology Information database (http://www.ncbi.nlm.nih.gov) together with our set (Figure 2) and separately (Technical Appendix Figure 2). Certain components, primarily mobile elements, were abundant within these plasmids: the average plasmid shared 500 contiguous bases with 58 of the other plasmids; however, median BLAST identity for this pairwise comparison was <12%, even when considering plasmids with the same β-lactamase, suggesting that both carbapenemases exist within a variety of plasmid configurations.

To visualize this comparison of carbapenemase plasmids, we generated a network diagram in which each node represented a plasmid and each line represented shared sequence between 2 plasmids (Figure 2, panel B). Node size and line width correlate to the number of nucleotides contained in the plasmid or sharing interaction. This visualization shows the abundant small, shared regions that exist between most plasmid pairs, represented as thin background lines. This visualization also highlights the larger shared regions that indicate highly similar plasmids, represented by the few wide lines. These outliers were often between pairs of plasmids encoding the same β-lactamase but were also observed between NDM-1 and KPC containing plasmids (maximum 79% of smaller plasmid length).

## Conclusions

Together, this evidence supports our hypothesis that strains and plasmids known to carry either carbapenemase also have access to the other. Given the similarity of carbapenemase-negative strains to those carrying KPC or NDM-1 and the high diversity of plasmids in which they can be found, we anticipate that global carbapenem usage will encourage HGT of both of these carbapenemases into additional strain and plasmid backgrounds. Because KPC and NDM-1 are poised to cross genetic and geographic boundaries, we recommend that hospitals routinely screen *Enterobacteriaceae* strains for both genes, even in regions where they are not yet endemic. We further advocate reduced carbapenem use to limit the selection for resistance against this vital antibiotic class.

Technical Appendix Phylogenetic analysis, sequence conservation between plasmids, and antibiotic drug susceptibility profiles of clinical isolates from Pakistan and the United States.
