# Supplementary material for: KPC and NDM-1 Genes in Related Enterobacteriaceae Strains and Plasmids from Pakistan and the United States
Source: Emerg Infect Dis. 2015 Jun;21(6):1034–7. doi: 10.3201/eid2106.141504 (PMC4451916; doi:10.3201/eid2106.141504)
Supplement: Technical Appendix — Phylogenetic analysis, sequence conservation between plasmids, and antibiotic drug susceptibility profiles of clinical isolates from Pakistan and the United States. [file 14-1504-Techapp-s1.pdf]

Publisher: CDC; Journal: Emerging Infectious Diseases
Article Type: Dispatch; Volume: 21; Issue: 6; Year: 2015; Article ID: 14-1504
DOI: 10.3201/eid2106.141504; TOC Head: Dispatch
Article DOI: http://dx.doi.org/10.3201/eid2106.141504

KPC and NDM-1 Genes in Related *Enterobacteriaceae* Strains and Plasmids from Pakistan and the United States

Technical Appendix

Methods and Materials

Sample Selection, Processing, and Phenotyping

We collected 450 Pakistani bacterial isolates (PH) initially recovered from de-identified clinical samples from urinary, blood stream, genitourinary, and wound infections collected between February 2012 and March 2013 at Pakistan Railway General Hospital, Rawalpindi, Pakistan, and the Pakistan Institute of Medical Sciences in Islamabad, Pakistan. These included all ESKAPE pathogen isolates available in the Pakistani hospital strain banks during the indicated collection period. From these 450 isolates, we chose a random subset of 55 isolates from the *Enterobacteriaceae* family (from a total of 195 *Enterobacteriaceae* in this collection) for phenotypic and genotypic analysis. We also selected 48 US *Enterobacteriaceae* isolates (WU) from banked, de-identified frozen stocks of *Escherichia coli, Klebsiella pneumoniae,* and *Enterobacter cloacae* at Barnes Jewish Hospital/Washington University School of Medicine in Saint Louis, Missouri, United States to have beta-lactam resistance and susceptibility phenotypes in similar proportions to the Pakistani *Enterobacteriaceae* isolates, with a particular focus on the meropenem resistance phenotype (protocols for growth and phenotyping are described below). The WU strains were originally isolated from urine, respiratory, bone, and bile specimens between January 2010 and June 2013. Of the 48 WU isolates, 23 isolates were chosen for genome sequencing to generally match the species distribution of the 55 PH isolates as well as their beta-lactam resistance profiles, with the exception of *Enterobacter aerogenes,* for which none were available in the WU collection. In total, 33 *Escherichia coli* (24 PH, 9 WU), 30 *Klebsiella pneumoniae* (19 PH, 11 WU), 9 *Enterobacter cloacae* (6 PH, 3 WU), and 6 *Enterobacter aerogenes* (all 6 PH) isolates were included for the whole genome sequencing

analysis (Technical Appendix Tables 1 and 2). We cultivated all isolates on MacConkey and sheep's blood agar (Hardy Diagnostics). We then grew single colonies in LB broth liquid culture for DNA extraction. We assessed each isolate for susceptibility to ampicillin, cefazolin, cefotetan, ceftazidime, ceftriaxone, cefepime, meropenem, ciprofloxacin, trimethoprim-sulfamethoxazole, gentamicin, doxycycline, and chloramphenicol by Kirby-Bauer disk diffusion according to Clinical and Laboratory Standards Institute guidelines and interpretive criteria (*1*). Prior to whole genome sequencing, the species identity of PH and WU isolates was determined with VITEK MS MALDI-TOF MS v2.0 knowledgebase (bioMerieux) as previously described (*2*,*3*). We then extracted total DNA using the Invitrogen Charge Switch gDNA Mini Bacteria kit per the manufacturer's protocol. We also extracted plasmid DNA from 11 KPC and 9 NDM-1 encoding isolates (as well as 3 CTX-M-15 encoding isolates), as determined by PCR and the genome sequencing, using the Qiagen Large Construct kit per the manufacturer's protocol. We included one non- *Enterobacteriaceae* plasmid preparation from an *Acinetobacter baumannii* isolate (PH), which had been identified to contain NDM-1 by PCR.

Illumina Library Preparation

We sheared 500ng of total DNA from each isolate to ~300 bp fragments fragments in nine rounds of shearing of ten minutes each on the BioRupter XL. In each round the power setting was 'H' and samples were treated for 30s and allowed to rest for 30s. Each sample was concentrated using the Qiagen MinElute PCR purification kit per the manufacturer's protocol. End Repair of the sheared DNA fragments was initiated with the addition of 2.5 μl of T4 DNA ligase buffer with 10mM ATP (NEB, B0202S), 1 μl of 1 mM dNTPs (NEB), 0.5 μl T4 Polymerase (NEB, M0203S), 0.5 μl T4 PNK (NEB M0201S), and 0.5 μl Taq Polymerase (NEB, M0267S). This mixture was incubated at 25°C for 30 min, then at 75°C for 20 min. Barcoded adapters were then added to the solution along with 0.8μl of T4 DNA ligase (NEB, M0202M), for the purpose of ligating the adapters to the DNA fragments. This solution was then incubated at 16ºC for 40min, then 65°C for 10min. At this point the adapter-ligated DNA was purified using the Qiagen MinElute PCR purification kit per the manufacturer's protocol.

The DNA fragments were then size selected on a 2% agarose gel in 1X TBE buffer stained with Biotium GelGreen dye (Biotium). DNA fragments were combined with 2.5uL 6X Orange loading dye before loading on to the gel. Adaptor-ligated DNA was extracted from gel

slices corresponding to DNA of 250-300bp using a QIAGEN MinElute Gel Extraction kit per the manufacturer's protocol. The purified DNA was enriched by PCR using 12.5µL 2X Phusion HF Master Mix and 1µL of 10µM Illumina PCR Primer Mix in a 25µL reaction using 1µL of purified DNA as template. DNA was amplified at 98°C for 30 seconds followed by 18 cycles of 98°C for 10 seconds, 65°C for 30 seconds, 72°C for 30 seconds with a final extension of 5min. at 72°C. Afterwards, the DNA concentration was measured using the Qubit fluorometer and 10nmol of each sample (up 106 per lane of sequencing) were pooled. Subsequently, samples were submitted for Illumina HiSeq-2500 Pair-End (PE) 101bp sequencing at GTAC (Genome Technology Access Center, Washington University in St. Louis) at 9pmol per lane.

Genome Sequence Assembly

All sequencing reads were de-multiplexed by barcode into separate genome bins. Reads were quality trimmed to remove adapter sequence and bases on either end with a quality score below 19. Any reads shorter than 31bp after quality trimming were not used in further analysis. The best reference sequence was chosen for each isolate or plasmid by mapping 10000 reads chosen randomly from that isolate against all reference genomes (from NCBI Genome, downloaded July 14th 2014) of the same species as the isolate (in the case of genomic DNA assembly) or against all plasmid sequences containing NDM-1, KPC, or CTX-M (in the case of plasmid DNA assembly). Reads were mapped using Bowtie 2 (*4*) (command: bowtie2 –x <reference_genome_index_name> -1 <forward_read_file> -2 <reverse_read_file> -q --phred33 --very-fast –I 100 –X 600 --no-discordant --no-mixed --no-unal --no-hd --no-sq --omit-sec-strand). The genome or plasmid against which the highest percentage of reads mapped was used as the reference sequence for that assembly. It was empirically determined that if this first mapping included fewer than 60% of the reads, then the assembly would be best done completely *de novo*. For isolates with >60% of reads matching a reference sequence, all reads were mapped to that sequence (command: bowtie2 –x <reference_genome_index_name> -1 <forward_read_file> -2 <reverse_read_file> -q --phred33 --very-sensitive-local -I 200 -X 1000 -S <sam_output>). Variants from the reference were called using samtools (commands: samtools view -buS <sam_file> | samtools sort -m 4000000000 - <sample_prefix> ### samtools index <bam_file> ### samtools mpileup -uD -f <reference_genome> <bam_file> | bcftools view -bcv -> <bcf_file> ### bcftools view <bcf_file>). The variant call format file was then filtered to

remove SNPs with a quality score lower than 70 or coverage greater than twice the average coverage expected per base. Custom scripts were then used to extract DNA sequences from the reference genome with > three independent reads, to create a fragment file of regions in the sample genome matching the reference genome modified with high-quality variant information.

De novo assembly of the reads from each isolate was completed using Velvet (*5*) (commands: velveth <output_directory> 51 -fastq -shortPaired <interleaved_reads> ### velvetg <output_directory> -ins_length 400 -exp_cov <kmer_coverage> -cov_cutoff <coverage_cutoff>). Kmer coverage was calculated as: total read coverage*0.50 (because the kmer length was approximately half the read length), and the coverage cutoff was calculated as the kmer coverage divided by eight. If a complete reference mapping was performed, then contigs from the *de novo* assembly and reference mapping were put in an additional velvet assembly step as long reads with the original reads files (commands: velveth <output_directory> 51 -fastq -shortPaired -separate <forward_reads> <reverse_reads> -fasta -long <de_novo_fragments> <reference_fragments> ### velvetg <output_directory> -ins_length 400 -clean yes -conserveLong yes -exp_cov <kmer_coverage> -cov_cutoff <coverage_cutoff> -scaffolding yes -long_mult_cutoff 0). Finally all fragments were collapsed on nucleotide identity using cd-hit (command: cd-hit-est -I <fragment_file> -o <collapsed_file> -d 0 -M 0). All fragments smaller than 500bp were partitioned to a separate file by a custom script. Plasmid sequences were assembled by this same method, with the sequences of all complete plasmids encoding, NDM-1, KPC, or CTX-M used as references.

ORF Prediction and Annotation

ORF prediction for each genome was performed separately using GeneMark (*6*) models based on the closest reference genome (command: gmhmmp -m <model_name> -o <outfile> -a <contig_name_file>). Each ORF was compared to three databases of profile hidden Markov models using HMMR (*7*): Pfam (command: hmmscan --cut_ga -o /dev/null --tblast <target_out_file> --domtblast <domain_out_file> <Pfam_database_file> <protein_input_file>), TIGRFAMs (command: hmmscan --cut_ga -o /dev/null --tblast <target_out_file> --domtblast <domain_out_file> <database_file> <protein_input_file>), and Resfams (dantaslab.wustl.edu/resfams/) (*8*) (command: hmmscan --cut_ga -o /dev/null --tblast <target_out_file> --domtblast <domain_out_file> <database_file> <protein_input_file>). All

functional annotations were concatenated into a single file by a custom script. 756 *E. coli* and 54 *K. pneumoniae* completed and draft genomes were downloaded from the National Center for Biotechnology Information (NCBI) on April 15th 2014, for the purpose of comparing to the isolate set. GeneMark models from the completed genomes were used to predict ORFs for those genomes, while for draft genomes models created from *E. coli* K12 MG1655 (for *E. coli* draft genomes) or *K. pneumoniae* KCTC 2242 (for *K. pneumoniae* draft genomes) were used for ORF prediction. All genome and plasmid sequences were deposited into NCBI (BioProject accession number: PRJNA261540).

In silico MLST

Multi-Locus Sequence Typing **(**MLST) profiles were downloaded from PubMLST (pubmlst.org). When an absolute MLST profile could not be identified for an organism (because of ambiguous bases or incomplete assembly of one or more loci) the remaining possible sequence types (ST) based on the incomplete information were identified. In all cases a strain could be identified as one of at most 19 ST using this methodology. MLST profiles were only applied to *Escherichia coli* and *Klebsiella pneumoniae* since the PubMLST database does not contain an *Enterobacter* table.

Core Genome Alignment

Command for whole genome alignment using mugsy (*9*): command: mugsy --directory <output_directory> --prefix <output_prefix> <genome_fasta_1> <genome_fasta_2> … <genome_fasta_N>. For *E. coli*, *K. pneumoniae*, and *E. cloacae* a single reference genome was included in the alignment to provide context (*E. coli* K12 MG1655, *K. pneumonaie* KCTC 2242, and *E. cloacae* ATCC 13047 respectively). Poorly aligned regions (i.e. plasmids or recombined regions, which could create noise in the phylogenetic signal) were removed using Gblocks (*10*) (command: Gblocks <input_file> -t=d -b3=24) leaving only the core genome alignment. Maximum likelihood trees made made by RaxML (*11*) (command: raxmlHPC-SSE3 -s <input_file> -n <output_file> -m GTRGAMMA -d -f a -N 100 -x 54321 -w <output_directory>) and FastTree (*12*) (command: FastTree -gtr -nt -gamma -nome <input_file> > <output_file>) were compared for agreement. When trees made by both methods were in agreement, the output from FastTree was used for visualization. Files were converted between various required formats by custom scripts.

Subspecies clades were defined as groups of branches descended from a common ancestor where no individual branch within the clade could have more than 0.005 substitutions per site. This definition yielded the same groupings as the *in silico* MLST described above, in all cases where all members of a clade could be assigned to a known ST (online Technical Appendix Figure 1, panels A,B).

Specific β-lactamase Identification

A BLAST database was constructed from the amino acid sequences of all β-lactamases cataloged in the Bush and Jacoby (*13*) database at www.lahey.org/Studies/ (accessed March 25th, 2014). Genes from our genomes annotated as β-lactamases were extracted and compared against this database by BLAST. Exact matches were then re-annotated with their specific β-lactamase name, while inexact matches were recorded as their closest hit plus an asterisk.

Plasmid Comparisons

We compared plasmid sequences by an all-against-all pairwise nucleotide BLAST alignment. For each pair of plasmids, we calculated the percentage of each plasmid that aligned at >99% identity. We then binned the percentages from each pairwise alignment into groups based on the defining β-lactamase of their query and subject plasmids. We also generated network diagrams from the pairwise BLASTs using custom Python scripts and Cytoscape (*14*), only including regions above 99% identity and over 500 bp.

Isolate Characteristics

The sampled Enterobacteriaceae isolates are phylogenetically diverse and include multiple examples of known pathogenic sequence types. We performed WGS of each isolate, totaling 33 *Escherichia coli* isolates, 30 *Klebsiella pneumoniae* isolates, 9 *Enterobacter cloacae* complex isolates, and 6 *Enterobacter aerogenes* isolates. We then used whole genome alignment of the core genomes of each species to reconstruct the phylogenetic relationships of each isolate at high resolution (Technical Appendix Figure 2). The species trees demonstrate that we sampled genomes from a variety of evolutionary clades as well as from multiple members of specific clades. They also demonstrate that clades could include isolates from both the United States and

Pakistan, allowing us to ignore the geographic variable and group the isolates by carbapenemase carriage for subsequent analyses. We also used housekeeping gene sequence from each isolate to perform in silico MLST, allowing us to compare our phylogenetic analysis to previously identified sequence types. We found that the clades on our tree include globally-disseminated pathogen sequence types, such as ST131 in *E. coli* and ST11 (single locus variant of ST258) in *K. pneumoniae*.

ST131 is noted for its virulence as well as for its frequent association with the CTX-M β-lactamases and fluoroquinolone resistance (*15–17*). Previous reports have found ST258, and closely related *K. pneumoniae*, to have relatively high rates of carbapenemase carriage (*18,19*). Despite the utility of MLST-based classification for large-scale epidemiological purposes, binning clinical isolates into sequence types masks genotypic and phenotypic variation due to HGT or single nucleotide polymorphisms, and therefore MLST cannot be used for fine-grained epidemiology or as an accurate predictor of antibiotic susceptibility. For example, two previous studies have shown that ST131 can be subdivided into three distinct lineages with different rates of antibiotic resistance (*15,16*). One of these studies found that the rapid global expansion of ST131 has been driven by the success of a specific subclone of ST131 that encodes fluoroquinolone resistant *gyrA* and *parC* alleles and CTX-M-15 (*16*), a characterization which fits 7 of our 11 ST131 isolates. We also identified a single ST131 isolate carrying KPC-2, which was resistant to all β-lactams tested. We also observed *K. pneumoniae* ST11 isolates carrying KPC-3, and others carrying NDM-1, which fits with reports characterizing ST11 as being highly common worldwide and frequently encoding carbapenemases (*18,19*).

Antibiotic Resistance Phenotypes

To establish the overall susceptibility profiles of each of our strains, we performed phenotypic tests using Kirby Bauer Disk diffusion in accordance with CLSI guidelines on all 78 clinical isolates against 12 antibiotics including 7 β-lactams (Technical Appendix Table 1). We found that 63% of all isolates were resistant to ciprofloxacin, a fluoroquinolone commonly used to treat urinary tract infections. We also found resistance to trimethoprim-sulfamethoxazole in 65% of isolates, and gentamicin, doxycycline, and chloramphenicol exhibited *in vitro* resistance in 45%, 54%, and 56% of isolates, respectively. In the β-lactams, we saw near universal resistance to ampicillin (96% of isolates) and variable resistance to the cephalosporins. A high

rate of resistance to meropenem was observed (31% of isolates), but this finding was not surprising since this was the property on which many of the isolates had been selected.

References

1. Clinical and Laboratory Standards Institute. Performance standards for antimicrobial susceptibly testing; twenty-third informational supplement. 2013.

2. Manji R, Bythrow M, Branda JA, Burnham CAD, Ferraro MJ, Garner OB, et al. Multi-center evaluation of the VITEK® MS system for mass spectrometric identification of non-*Enterobacteriaceae* Gram-negative bacilli. Eur J Clin Microbiol Infect Dis. 2014 2014/03/01;33(3):337-46. PubMed http://dx.doi.org//10.1007/s10096-013-1961-2

3. Richter SS, Sercia L, Branda JA, Burnham CAD, Bythrow M, Ferraro MJ, et al. Identification of *Enterobacteriaceae* by matrix-assisted laser desorption/ionization time-of-flight mass spectrometry using the VITEK MS system. Eur J Clin Microbiol Infect Dis. 2013 2013/12/01;32(12):1571-8. PubMed http://dx.doi.org//10.1007/s10096-013-1912-y

4. Langmead B, Salzberg SL. Fast gapped-read alignment with Bowtie 2. Nat Methods. 2012;9:357–9. PubMed http://dx.doi.org/10.1038/nmeth.1923

5. Zerbino DR, Birney E. Velvet: algorithms for de novo short read assembly using de Bruijn graphs. Genome Res. 2008;18:821–9. PubMed http://dx.doi.org/10.1101/gr.074492.107

6. Borodovsky M, Lomsadze A. Gene identification in prokaryotic genomes, phages, metagenomes, and EST sequences with GeneMarkS suite. Current protocols in bioinformatics / editoral board, Andreas D Baxevanis [et al]. 2011;Chapter 4:Unit 4.5.1-17.

7. Eddy SR. A new generation of homology search tools based on probabilistic inference. Genome Inform. 2009;23:205–11. PubMed http://dx.doi.org/10.1142/9781848165632_0019

8. Gibson MKFK, Dantas G. Improved annotations of antibiotic resistance functions reveals microbial resistomes cluster by ecology. ISME. 2014. PubMed http://dx.doi.org//10.1038/ismej.2014.106

9. Angiuoli SV, Salzberg SL. Mugsy: fast multiple alignment of closely related whole genomes. Bioinformatics. 2011;27:334–42. PubMed http://dx.doi.org/10.1093/bioinformatics/btq665

10. Talavera G, Castresana J. Improvement of phylogenies after removing divergent and ambiguously aligned blocks from protein sequence alignments. Syst Biol. 2007;56:564–77. PubMed http://dx.doi.org/10.1080/10635150701472164

11. Stamatakis A. RAxML Version 8: A tool for phylogenetic analysis and post-analysis of large phylogenies. Bioinformatics. 2014 January 21, 2014. PubMed http://dx.doi.org/10.1093/bioinformatics/btu033

12. Price MN, Dehal PS, Arkin AP. FastTree 2–approximately maximum-likelihood trees for large alignments. PLoS ONE. 2010;5:e9490. PubMed http://dx.doi.org/10.1371/journal.pone.0009490

13. Bush K, Jacoby GA. Updated functional classification of beta-lactamases. Antimicrob Agents Chemother. 2010;54:969–76. PubMed http://dx.doi.org/10.1128/AAC.01009-09

14. Smoot ME, Ono K, Ruscheinski J, Wang P-L, Ideker T. Cytoscape 2.8: New features for data integration and network visualization. Bioinformatics. 2011;27:431–2 dx.doi.org//10.1093/bioinformatics/btg675. PubMed http://dx.doi.org/10.1093/bioinformatics/btq675

15. Petty NK, Ben Zakour NL, Stanton-Cook M, Skippington E, Totsika M, Forde BM, et al. Global dissemination of a multidrug resistant *Escherichia coli* clone. Proc Natl Acad Sci U S A. 2014;111:5694–9. PubMed http://dx.doi.org/10.1073/pnas.1322678111

16. Price LB, Johnson JR, Aziz M, Clabots C, Johnston B, Tchesnokova V, et al. The epidemic of extended-spectrum-beta-lactamase-producing *Escherichia coli* ST131 is driven by a single highly pathogenic subclone, H30-Rx. MBio. 2013;4:e00377–13. PubMed http://dx.doi.org/10.1128/mBio.00377-13

17. Rogers BA, Sidjabat HE, Paterson DL. *Escherichia coli* O25b-ST131: a pandemic, multiresistant, community-associated strain. J Antimicrob Chemother. 2011;66:1–14. PubMed http://dx.doi.org/10.1093/jac/dkq415

18. Cuzon G, Naas T, Truong H, Villegas MV, Wisell KT, Carmeli Y, et al. Worldwide diversity of *Klebsiella pneumoniae* that produce beta-lactamase blaKPC-2 gene. Emerg Infect Dis. 2010;16:1349–56. PubMed http://dx.doi.org/10.3201/eid1609.091389

19. Pena I, Picazo JJ, Rodriguez-Avial C, Rodriguez-Avial I. Carbapenemase-producing *Enterobacteriaceae* in a tertiary hospital in Madrid, Spain: high percentage of colistin resistance among VIM-1-producing *Klebsiella pneumoniae* ST11 isolates. Int J Antimicrob Agents. 2014;43:460–4. PubMed http://dx.doi.org/10.1016/j.ijantimicag.2014.01.021

Publisher: CDC; Journal: Emerging Infectious Diseases
Article Type: Dispatch; Volume: 21; Issue: 6; Year: 2015; Article ID: 14-1504
DOI: 10.3201/eid2106.141504; TOC Head: Dispatch

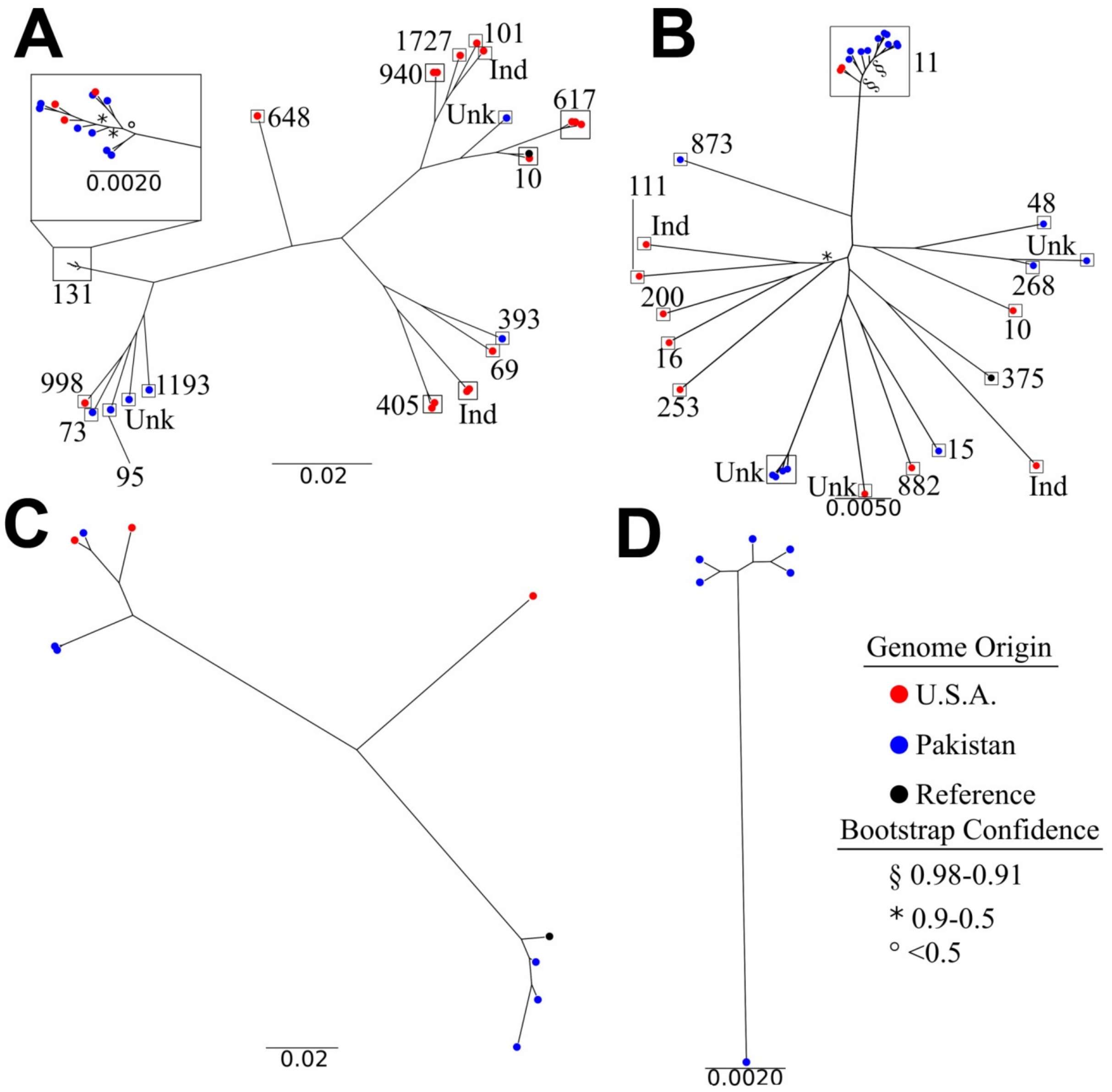

Technical Appendix Figure 1. Phylogenetic trees for isolates from samples collected in Pakistan and the United States. Trees are separated by species, A) *Escherichia coli*, B) *Klebsiella pneumoniae*, C) *Enterobacter cloacae*, and D) *Enterobacter aerogenes*, but not rooted. Bootstrap values are 1 for each branch unless otherwise noted. For each species ≈50% of the genome was determined to be core, and was used for phylogenetic inference. Scale bars indicate the nucleotide substitutions per site. In a) and b) sequence types (ST) as determined by *in silico* multilocus sequence typing are indicated by boxes grouping members of the same ST together. Unk = ST does not correspond to any reported in pubMLST, Ind = exact ST could not be determined due to sequencing error. Reference genomes included for *E. coli* (K12 MG1655), *K. pneumoniae* (KCTC 2242), and *E. cloacae* (ATCC 13047) on their respective trees.

Publisher: CDC; Journal: Emerging Infectious Diseases
Article Type: Dispatch; Volume: 21; Issue: 6; Year: 2015; Article ID: 14-1504
DOI: 10.3201/eid2106.141504; TOC Head: Dispatch

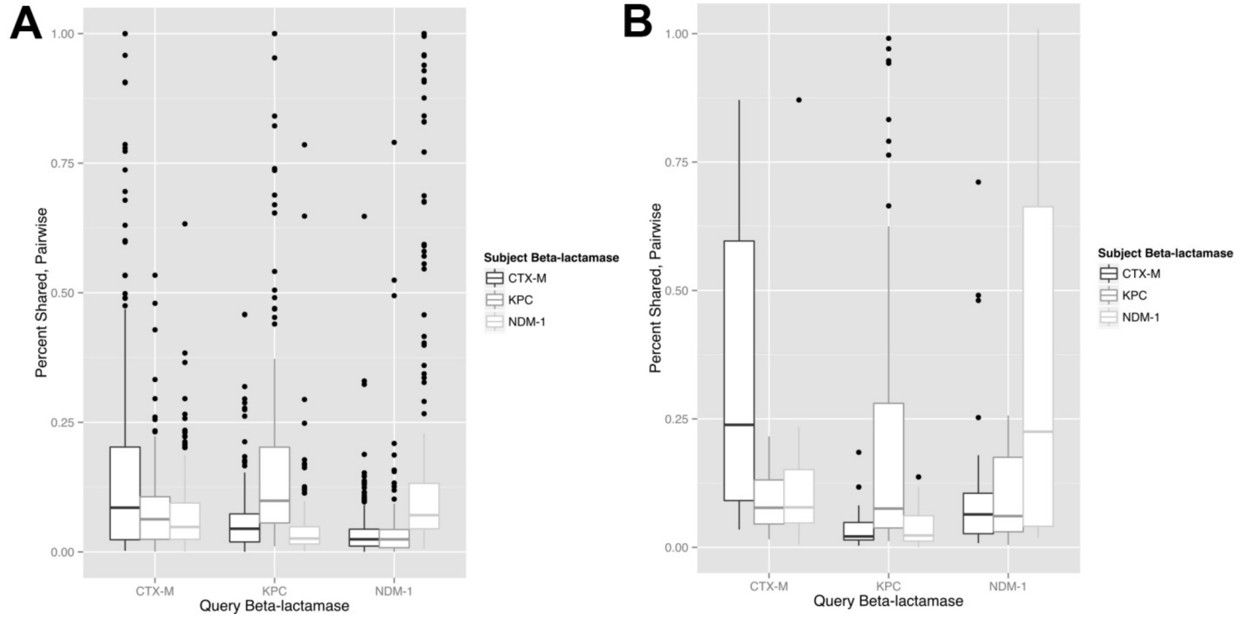

Technical Appendix Figure 2. Sequence conservation between plasmids containing NDM-1, KPC, or CTX-M β-lactamases. All plasmids from A) NCBI and B) this study that contained a NDM-1, KPC, or CTX-M β-lactamase were analyzed by all-against-all BLAST. Plasmid interactions were defined by the percentage of the query plasmid conserved (at >99% identity) in the subject plasmid. Plasmid interactions were plotted based on the defining β-lactamase of their query and subject plasmids.

Technical Appendix Table 1. Antibiotic drug susceptibility profiles of clinical isolates from Pakistan and the United States*

Species, no.	Location	Phenotype	AM	CZ	CTT	CAZ	CRO	FEP	MEM	CIP	SXT	GM	D†	C†
E. coli, 9	U.S.A	R	78%	67%	22%	44%	44%	44%	44%	56%	33%	22%	33%	33%
		I	0%	11%	22%	0%	0%	0%	0%	0%	0%	0%	0%	11%
		S	22%	22%	56%	56%	56%	56%	56%	44%	67%	78%	11%	0%
E. coli, 24	Pakistan	R	96%	83%	13%	38%	63%	21%	0%	67%	75%	38%	67%	33%
		I	4%	17%	0%	13%	0%	21%	0%	0%	0%	0%	17%	4%
		S	0%	0%	88%	50%	38%	58%	100%	33%	25%	63%	17%	63%
K. pneumoniae, 11	U.S.A	R	100%	36%	0%	36%	36%	36%	36%	27%	36%	9%	18%	27%
		I	0%	0%	36%	0%	0%	0%	0%	0%	9%	0%	0%	0%
		S	0%	64%	64%	64%	64%	64%	64%	73%	55%	91%	18%	9%
K. pneumoniae, 19	Pakistan	R	100%	68%	21%	63%	63%	42%	16%	63%	68%	58%	32%	58%
		I	0%	5%	0%	0%	0%	16%	0%	11%	5%	0%	21%	11%
		S	0%	26%	79%	37%	37%	42%	84%	26%	26%	42%	47%	32%
E. cloacae, 3	U.S.A	R	100%	100%	100%	100%	100%	100%	100%	67%	67%	33%	33%	33%
		I	0%	0%	0%	0%	0%	0%	0%	0%	0%	33%	33%	33%
		S	0%	0%	0%	0%	0%	0%	0%	33%	33%	33%	33%	33%
E. cloacae, 6	Pakistan	R	83%	100%	67%	100%	100%	83%	67%	83%	83%	100%	50%	83%
		I	0%	0%	0%	0%	0%	17%	0%	17%	0%	0%	17%	0%
		S	0%	0%	33%	0%	0%	0%	33%	0%	17%	0%	33%	17%
E. aerogenes, 6	Pakistan	R	100%	100%	17%	100%	100%	100%	100%	100%	100%	67%	83%	100%
		I	0%	0%	83%	0%	0%	0%	0%	0%	0%	0%	17%	0%
		S	0%	0%	0%	0%	0%	0%	0%	0%	0%	33%	0%	0%

*AM, ampicillin; CZ, cefazolin; CTT, cefotetan; CAZ, ceftazidime; CRO, ceftriaxone;FEP, cefepime; MEM, meropenem; CIP, ciprofloxacin; SXT, trimethoprim-sulfamethoxazole; GM , gentamicin; D, doxycycline; C, chloramphenicol; R, Resistant; I, Intermediate; S, Susceptible.
†For USA *E.coli* and *K. pneumoniae* in doxycycline and chloramphenicol selections, n=4.

Technical Appendix Table 2. Assembly metrics for whole genome assemblies

Species	Genome	Number of contigs	N50	Largest contig	Total nucleotides
Escherichia coli	PH100	1288	35053	385222	6309191
Escherichia coli	PH101–2	404	31119	177520	4857948
Escherichia coli	PH105	288	55979	220618	5045926
Escherichia coli	PH108	754	12427	95430	4930975
Escherichia coli	PH114	280	68389	450011	5902296
Escherichia coli	PH118	1028	13550	93913	5073129
Escherichia coli	PH129	235	76630	188170	5470109
Escherichia coli	PH135	156	87914	545356	4864787
Escherichia coli	PH141	656	14806	87255	4469903
Escherichia coli	PH143	355	33581	145252	4875919
Escherichia coli	PH151–2	368	41647	143184	5066089
Escherichia coli	PH156–1	720	24256	203811	5434228
Escherichia coli	PH18	357	39599	126851	5026978
Escherichia coli	PH20	513	20802	122933	4967604
Escherichia coli	PH31	394	40363	219779	5077941
Escherichia coli	PH39	465	28063	96484	4695860
Escherichia coli	PH51	1108	13989	85055	4988648
Escherichia coli	PH5	193	185476	496282	5367128
Escherichia coli	PH85	331	45973	149681	5118463
Escherichia coli	PH90	475	31325	258054	5169559
Escherichia coli	PH92–1	738	19526	90560	5313215
Escherichia coli	PH93	487	31876	685039	5927902
Escherichia coli	PH94	325	40628	218502	4960477
Escherichia coli	PH98	401	28050	272240	4791597
Escherichia coli	WU31	502	21374	148889	4858305
Escherichia coli	WU32	329	43395	166954	5598148
Escherichia coli	WU33	487	25419	139243	4975544
Escherichia coli	WU34	278	67976	229510	5206081
Escherichia coli	WU35	267	51428	235055	4846364
Escherichia coli	WU40	145	202444	388283	5052711
Escherichia coli	WU43	193	91836	346403	5033909
Escherichia coli	WU44	196	92332	610815	5473063
Escherichia coli	WU45	332	40224	155439	4990710
Klebsiella pneumoniae	PH102	2205	3247	29455	4976237
Klebsiella pneumoniae	PH10	192	129071	506496	5877659
Klebsiella pneumoniae	PH11	224	109166	648719	5657202
Klebsiella pneumoniae	PH124	238	66778	202869	5515528
Klebsiella pneumoniae	PH12	317	46831	180775	5530414
Klebsiella pneumoniae	PH139	263	92250	433917	5458209
Klebsiella pneumoniae	PH150–2	487	40719	311295	5568393
Klebsiella pneumoniae	PH152	354	74725	275992	5863335
Klebsiella pneumoniae	PH24–1	181	209112	601209	5541053
Klebsiella pneumoniae	PH25	439	51744	186984	5437740
Klebsiella pneumoniae	PH28–1	302	72750	480318	5876774
Klebsiella pneumoniae	PH38–1	178	243482	614325	6135768
Klebsiella pneumoniae	PH40	471	43868	232550	6212797
Klebsiella pneumoniae	PH44	251	133296	479093	6297207
Klebsiella pneumoniae	PH49–2	273	84383	550379	6883299
Klebsiella pneumoniae	PH72	2195	13163	144014	7250407
Klebsiella pneumoniae	PH73	997	11652	91567	5804995
Klebsiella pneumoniae	PH88	150	289170	512144	5630043
Klebsiella pneumoniae	PH9	927	12231	72933	5207334
Klebsiella pneumoniae	WU10	179	117943	480943	5928719
Klebsiella pneumoniae	WU12	1131	12084	59345	5227362
Klebsiella pneumoniae	WU18	961	14636	96067	5664085
Klebsiella pneumoniae	WU21	372	67952	249904	5556247
Klebsiella pneumoniae	WU23	142	208533	460529	5958526
Klebsiella pneumoniae	WU2	407	55286	345910	6330941
Klebsiella pneumoniae	WU3	705	94321	408241	6853166
Klebsiella pneumoniae	WU6	297	104936	426673	5919869
Klebsiella pneumoniae	WU7	227	79691	298601	5818526
Klebsiella pneumoniae	WU8	377	64077	729491	7506385
Klebsiella pneumoniae	WU9	141	120728	292977	5557123
Enterobacter aerogenes	112–2	90	177700	566534	5153448
Enterobacter aerogenes	PH113	450	22178	97515	4695038
Enterobacter aerogenes	PH134	274	107942	399309	5126469
Enterobacter aerogenes	PH138	776	30613	144769	9901518

Species	Genome	Number of contigs	N50	Largest contig	Total nucleotides
Enterobacter aerogenes	PH63	225	47345	184904	5211429
Enterobacter aerogenes	PH84–2	226	61644	336177	5307861
Enterobacter cloacae	PH23	179	83227	473778	5536166
Enterobacter cloacae	PH24–2	1238	20166	178617	8756501
Enterobacter cloacae	PH112–1	621	175912	517857	9155709
Enterobacter cloacae	PH125	1221	45282	215620	6004095
Enterobacter cloacae	PH158	552	34922	172567	9711730
Enterobacter cloacae	PH82	389	29307	134776	4974173
Enterobacter cloacae	WU26	538	46418	208322	5369428
Enterobacter cloacae	WU27	315	45300	155565	5251409
Enterobacter cloacae	WU29	457	24686	86433	4888311
Average	NA	490	67666	285311	5664479

Technical Appendix Table 3. Assembly metrics for plasmid assemblies

Species	Genome	Number of contigs	N50	Largest contig	Total nucleotides
Acinetobacter baumannii	PH147_2	98	3072	7683	181370
Escherichia coli	WU31	41	16473	48183	214615
Escherichia coli	WU32	86	11069	31992	233185
Escherichia coli	WU33	87	9555	47149	168182
Klebsiella pneumoniae	PH11	53	19073	48703	230288
Klebsiella pneumoniae	PH88	39	15980	48463	218205
Klebsiella pneumoniae	WU13	6	12943	12943	18694
Klebsiella pneumoniae	WU14	4	12976	12976	18501
Klebsiella pneumoniae	WU17	374	4432	60538	328889
Klebsiella pneumoniae	WU18	40	18017	43330	221015
Klebsiella pneumoniae	WU19	37	22152	87768	315122
Enterobacter aerogenes	PH112_2	80	19156	52826	450788
Enterobacter aerogenes	PH113	17	5703	19905	59909
Enterobacter aerogenes	PH134	22	4168	9715	48836
Enterobacter aerogenes	PH138	171	8795	35183	466555
Enterobacter aerogenes	PH63	37	29896	44891	282470
Enterobacter cloacae	PH23	111	12334	42017	516173
Enterobacter cloacae	PH24_2	95	15201	41616	562271
Enterobacter cloacae	PH82	206	9384	37900	641720
Average	NA	84	13178	38620	272463
